# Selection of Lactic Acid Bacteria Species and Strains for Efficient Trapping of *Drosophila suzukii*

**DOI:** 10.3390/insects12020153

**Published:** 2021-02-11

**Authors:** Amani Alawamleh, Gordana Ðurović, Giuseppe Maddalena, Raffaele Guzzon, Sonia Ganassi, Maaz Maqsood Hashmi, Felix Wäckers, Gianfranco Anfora, Antonio De Cristofaro

**Affiliations:** 1 Department of Agricultural, Environmental and Food Sciences, University of Molise, Via De Sanctis, 86100 Campobasso, Italy; amaniawamleh@yahoo.com (A.A.); peppemad@hotmail.com (G.M.); sonia.ganassi@gmail.com (S.G.); decrist@unimol.it (A.D.C.); 2 Biobest Group NV, Ilse Velden, 2260 Westerlo, Belgium; valerianaof@gmail.com (G.Ð.); Felix.Waeckers@biobestgroup.com (F.W.); 3 Research and Innovation Centre, Fondazione Edmund Mach, Via Edmund Mach 1, 38098 San Michele all’Adige, Italy; 4 Technology Transfer Centre, Fondazione Edmund Mach, 38098 San Michele all’Adige, Italy; raffaele.guzzon@fmach.it; 5 Consiglio Nazionale Della Ricerche, Instituto di Ricerca Sugli Ecosistemi Terrestri, Via Guglielmo Marconi 2, 05010 Porano, Italy; maazmh@hotmail.com; 6 Centre Agriculture Food Environment (C3A), University of Trento, Via Edmund Mach 1, 38098 San Michele all’Adige, Italy

**Keywords:** lactic acid bacteria, *Oenococcus*, food bait, spotted wing drosophila

## Abstract

**Simple Summary:**

The spotted wing drosophila (SWD) is an invasive fruit fly that causes serious economic damage to many fruit crops. Monitoring is the first step for any management program to determine the characteristics of a pest. For this purpose, there are no efficient baits registered to date. Certain bacteria release chemical compounds that attract SWD. We studied the bacterial impact on the enhanced attractiveness of a commercial bait (Droskidrink^®^) under field and laboratory conditions. At first, *Oenococcus oeni* belonging to lactic acid bacteria (LAB) was found to release chemical compounds that were highly attractive for SWD. The attractiveness of Droskidrink^®^ bait was increased by *O. oeni* culture, resulting in a higher capture rate of SWD in traps. Therefore, our findings suggest the use of the bacterial culture inside the commercial SWD baits. The use of these kinds of baits can minimize the risk of pest outbreaks in fruit orchards in both domestic and wild environments. Our pest management approach is farmer-friendly in all aspects, as well as the food sector.

**Abstract:**

(1) Monitoring of *Drosophila suzukii* is based on the use of effective traps and baits. The current baits are insufficient to provide efficient monitoring. The use of bacteria as bio-catalyzers to produce bioactive volatiles may improve flies’ attraction. Thus, we conducted this work to improve Droskidrink^®^ bait’s attractiveness using lactic acid bacteria. (2) Different baits that were based on the use of Droskidrink^®^ were assessed for flies’ attraction in a Droso-Trap^®^ in a vineyard. *Oenococcus oeni*, *Pediococcus* spp., and *Lactobacillus* spp. were used. The performance of the most attractive species, *O. oeni*, inoculated into Droskidrink^®^ was assessed in laboratory tests. The responses of female flies to volatiles produced by Droskidrink^®^ with *O. oeni* strains were recorded by electroantennography. (3) Preliminary field assessment of baits recorded *O. oeni* as the most attractive species. Three strain groups showed adaptation to test conditions. Volatiles extracted by the headspace of baits inoculated with *O. oeni*, elicited electroantennographic responses from fly antennae. (4) Droskidrink^®^ inoculated with *O. oeni* is a highly attractive bait for monitoring. These findings will be useful for improving the attractiveness of *D. suzukii* commercial baits based on the utilization of LAB volatiles in a strain-dependent manner.

## 1. Introduction

*Drosophila suzukii* Matsumura (Diptera: Drosophilidae) is an invasive fruit fly of Asian origin that rapidly invaded Europe, causing great losses in soft fruit production [1,2,3,4]. The rapid invasive spread of *D. suzukii* across Europe expanded to temperate/cool climates, causing severe damage to fruit crops and posing an economic threat to the small soft fruit industries [3]. Fruit damage is caused by larva feeding and development within the infested fruits, making them non-saleable [4]. *D. suzukii* is a highly polyphagous pest that infests a broad range of soft-skinned fruits, i.e., berry fruits blackberry, raspberry, blueberry, strawberry, and grapes [2]. 

The management of targeted pests is mainly implemented with conventional chemical insecticides. Among the registered insecticides, organophosphates, spinosyns and pyrethrins, in timely applications, can provide an adequate level of control [5,6,7]. At present, chemical control methods often show limitations and failures due to pest population density, and the specific social and agronomical context. Among environmentally safe approaches, those based on the interferences with insect communication often provide efficient control, i.e., mass trapping and attract and kill [8,9]. On the other hand, *D. suzukii* populations could be reduced by exploiting biocontrol agents (fungi, bacteria, and viruses) and other natural enemies of this pest, such as parasitoids and predators [10,11,12,13]. However, the evaluation of these control methods in the laboratory and in field assays and their development for commercial use are still ongoing.

Therefore, *D. suzukii* monitoring, as a part of pest management, is the key to controlling this pest. It assists in the detection of *D. suzukii* in host crops and provides information about feeding patterns, behavior throughout the year, and population dynamics. Hence, it is important to use the best available traps and baits to obtain the most adequate monitoring. Primarily, *D. suzukii* population monitoring is being implemented by using differentially shaped and colored traps baited with fermentation products such as apple cider vinegar, wine, or yeast/sugar as attractive lures [14,15,16,17,18,19,20,21]. At present, fermentation products are not adequately selective and effective for *D. suzukii* monitoring [20], and no efficient monitoring tools have been developed yet. Despite that, it has been demonstrated that the attractiveness of food baits can be increased by the addition of different bacterial species. In this regard, the investigation of volatile organic compounds produced by bacterial fermentation identified the key compounds for effective fly attraction. Such attractiveness has been investigated in behavioural bioassays, along with the chemical characterization of the volatiles profile. Strains and species of symbiotic acetic acid bacteria commonly found in *D. suzukii* Italian populations (*Acetobacter*, *Gluconobacter*, and *Komagataeibacter*) were tested with an olfactometer. Female flies showed a significant attraction to some strains of *Gluconobacter* and *Komagataeibacter* species, whose volatile compounds were proposed as a useful tool for developing sustainable control strategies [22].

Lactic acid bacteria (LAB) are widespread microorganisms which can be found in any environment rich in carbohydrates, such as plants and fermented foods. They are Gram-positive and acid-tolerant microorganisms that grow anaerobically. LAB include several species, i.e., the genus *Lactobacillus*, as well as the genera *Pediococcus*, *Leuconostoc*, *Streptococcus*, and *Oenococcus.* The fast-growing characteristics of LAB strains and their metabolic activities are the key to LAB’s benefits and applications [23]. 

The primary LAB metabolic activity is the degradation of carbohydrates into different compounds, mainly lactic acid through the fermentation process [24]. However, they share several unique biochemical characteristics when compared to other bacterial groups (e.g., high malolactic activity, production of volatile compounds, growth rate) [25]. *Oenococcus oeni* (Garvie) is the species that best adapts to the harsh wine conditions, due to its ability to tolerate low pH and high concentrations of ethanol and sulfites [26]. These LAB characteristics distinguish them from other bacterial species, making them good candidates for a more attractive and selective food bait.

The LAB’s ability to produce bioactive volatiles attracting fruit Drosophila flies was evaluated in previous studies. The *L. brevis* and *L. plantarum* species were identified from *Drosophila melanogaster* (Meigen) larval gut, and their emitted volatile compounds were evaluated for fly attraction. Moreover, larval and adult flies showed a significant attraction to volatiles emanating from food substrates that have been occupied by larvae, indicating that flies rely on microbial volatiles for long distance attraction to suitable feeding sites [27].

Additionally, *D. melanogaster* exhibited behavioral preferences towards gut microbes. Both adults and larvae were attracted to volatile compounds associated with the fly microbiome, i.e., *L. plantarum* in choice assays [28]. Similarly, symbiotic LAB species were identified in *D. suzukii* gut, i.e., *L. plantarum* and *L. brevis* [29]. *D. suzukii* attraction towards volatiles emitted by LAB species has not been assessed yet. There is a need to investigate the use of LAB species as a source of attraction for *D. suzukii* flies and their subsequent potential application as components of a more attractive food bait. 

The current study attempts to fill numerous gaps in knowledge regarding the attractive effect of volatiles emitted by LAB species on *D. suzukii* fly preferences which will be helpful for developing effective tools for *D. suzukii* monitoring. Therefore, we decided to improve the attractiveness of commercially available Droskidrink^®^ food bait using active cultures of LAB species. In this study, we aimed to evaluate the attractiveness of different LAB species and strains for *D. suzukii* flies under field and laboratory conditions. The data obtained contribute to improving the food baits that are currently used to monitor *D. suzukii* and to developing new tools for efficient pest management, focusing on the exploitation of bioactive volatiles emitted by LAB strains. 

## 2. Materials and Methods

### 2.1. Field Assessment of the Attractiveness of Droskidrink^®^ Food Baits Inoculated with Different Lactic Acid Bacteria

A preliminary field experiment to compare the attractiveness of different food baits for *D. suzukii* flies was conducted in a commercial vineyard var. Teroldego in San Michele all’Adige, Trento, Italy. The commercial food bait Droskidrink^®^ (DD) (Prantil, Trento, Italy) was used in all bait types. DD comprises a mixture of apple cider vinegar (75%), red wine (25%), and 20 g/L of brown cane sugar [30]. The tested LAB including *Oenococcus oeni*, *Lactobacillus* spp., and *Pediococcus* spp. were selected from the bacterial culture collection of Fondazione Edmund Mach laboratories, San Michele all’Adige, Italy and inoculated in the commercial food bait.

The species were previously isolated from traditional Italian wine during the early phase of malolactic fermentation and identified based on gene sequencing [31]. LAB species were used as catalyzers of the production of bioactive compounds to *D. suzukii*, and their performance was assessed based on the biochemical changes in the DD bait. In this experiment, 10 types of food bait were used with different compositions at different pH values, as indicated in Table 1.

Droso-Trap^®^ (Biobest, Westerlo, Belgium) was deployed for field trapping. In brief, the trap designed for trapping Drosophila flies consists of a transparent lid with a wire hanger and a red plastic base containing three inlet tubes, each with seven holes with a 5 mm diameter for fly entry. The pH of the baits inoculated with bacterial strains was adjusted to 4.0 to ensure adequate bacterial growth. A drowning solution of DD was placed within the trap cup. Each trap contained 200 mL of liquid bait and a drop of Triton^®^ X-100 (Sigma-Aldrich Co., St. Louis, MO, USA) to reduce the liquid bait surface tension and promote the capture and submerging of flies. 

Bait type H was pasteurized to inhibit any microbial growth in the liquid bait. Bait type I was mixed with 10 mL/L of cycloheximide solution 0.01% (Oxoid, Cheshire, UK) to prevent yeast contamination. Bait type L was mixed with tetracycline (antibiotic) to inhibit bacterial growth in the liquid bait. The nominal concentration of bacterial species inoculated into each bait was adjusted to 10^6^ CFU/mL. The traps were placed in shady spots, 1 m above the ground at the level of the grapes. The randomized complete block design was applied in three different blocks with three replications each (Figure S1). All traps were maintained in the field for a seven-week period and checked weekly. Liquid baits were examined in the laboratory, captures of *D. suzukii* flies were recorded, and the liquid bait was replaced once a week. The numbers of *D. suzukii* flies were analyzed with a Poisson generalized linear model (GLM) [32]. 

The experiment included three trials that were conducted sequentially during the summer of 2013, using a different number of treatments in each trial. Each trial comprised three replicates where 6, 7, and 10 sample traps were placed in the first, second, and third trial, respectively. All trials were carried out in the same vineyard, adjacent to the forest area. The first trial focused on comparing DD baits inoculated with LAB strains i.e., A, B, C, D, E, and F baits. In the second trial, we focused on comparing the first six tested baits against the commercial version of DD (bait type G). Finally, we compared all previously tested baits with DD baits of different compositions, i.e., H, I, and L.

### 2.2. Assessment of The Performance of Oenococcus oeni Strains in Droskidrink^®^ Food Bait

Based on the findings of the preliminary field experiment (see Results section), *O. oeni* was selected as the most suitable bio-catalyzer to improve the attractiveness of DD. Therefore, fourteen strains of *O. oeni* were tested to determine their viability and fermentative metabolism in a standard medium for LAB. Strains isolated from Italian wines were obtained from the bacterial culture collection of Fondazione Edmund Mach laboratories, San Michele all’Adige, Italy. The selected strains showed high resistance to wine-limiting factors (low pH, high ethanol concentration, and low fermentation temperature) and performed a reliable malolactic fermentation in previous work [33].

All strains were cultured in modified MRS broth medium (MRSm) (Oxoid, Milan, Italy) in a 96 micro volume (200 µL) plate (Starstedt, Germany) at 25 °C. Thereafter, the MRSm was used to assay the bacterial growth at the main values of the Droskidrink^®^ parameters (pH value 4.0, acetic acid concentration 45 g/L, ethanol content 4% (*v*/*v*)) that would act as limiting factors for the metabolic performance of *O. oeni* strains. In this case, strains were tested at pH value 4.0, acetic acid concentration 45 g/L, ethanol content 4% (*v*/*v*), and incubation temperature 15 °C, all likely encountered during the field trapping. In each test, one DD parameter value was assessed with standard values of other parameters. In this experiment, three technical replicates and three biological replicates were used. Optical density (OD) measurements of bacterial cultures were performed in a PowerWave HT Microplate Spectrometer (BioTek, Winooski, VT, USA) with a Costar Flat Bottom 96-well plate with lid and 200 µL per well. Absorbance was measured at wavelength 480 nm and temperature 25 °C and the mean of three readings was taken. The increase in OD at 480 nm was measured. The OD at 480 nm of MRSm was used as blank. The incubation time was adjusted according to the methods recommended by the International Organization of Viticulture and Wine (OIV).

### 2.3. Electroantennography Responses of Drosophila suzukii Females to Volatile Collection of Oenococcus oeni Strains in Droskidrink^®^ Food Bait

On the basis of the assessment of the performance of fourteen *O. oeni* strains in DD bait (see Results section), three strains were selected and used to determine the electrophysiological response of *D. suzukii* flies to volatile compounds released during the fermentation process. Then, the antennal activity responses of mated females (*n* = 5) to the volatile compounds released by the three selected *O. oeni* strains (strain 2, strain 12, and strain 13) and the reference strain MRI 10000 were recorded by using a standard electroantennography (EAG) apparatus (Syntech). 

Experimental flies were collected from a laboratory colony that had already been established from collected fly populations in Trento Province, Italy. The colony was reared on a standard cornmeal-based artificial diet and maintained at 24 ± 1 °C, 65 ± 5% relative humidity and (16:8 L:D) photoperiod. Volatiles were collected with the closed loop stripping approach (CLSA) [34]. A 50 mL DD sample was poured into a glass jar with a plastic lid with two small openings. Both openings were connected with tubes in a circuit through the jar, where one tube was connected to a CLSA carbon filter (Brechbühler AG, Schlieren, Switzerland) while the other one was connected to the miniature 12 V vacuum graphite pump (Fürgut GmbH, Tannheim, Germany) to circulate the air through the jar. After 60 min of air flux, volatiles concentrated in the CLSA filter were eluted with 100 µL of dichloromethane (J.T. Baker, Deventer, Holland). 

Each eluted sample represented a stimulus. Each stimulus was prepared by adsorbing aliquots of the solution (25 µL) on filter paper 1.5 cm^2^ and inserting them into a Pasteur pipette. The Pasteur pipettes were closed on the thinner side with a 1 mL blue tip. The solvent was allowed to evaporate for 10 min before starting the experiment. Three control pipettes, including an empty pipette, a pipette filled with paraffin oil solvent, and a pipette filled with dichloromethane solvent, served as blanks. Before and after each recording, the responses of *D. suzukii* females to three reference stimuli (dichloromethane solvent, 1-hexanol and 2-hexanal) were recorded. These reference compounds are well known plant volatiles and significantly elicited antennal responses in *D. suzukii* [35]. 

Each stimulus was prepared by absorbing 25 μL of dichloromethane solution at a concentration of 1 μg/μL on the filter paper. A glass capillary indifferent electrode filled with Kaissling solution was introduced into the detached fly’s head. The different electrode was a similar capillary, connected to the distal end of antenna. Thereafter, the stimulus output was directly delivered to the antenna with the Pasteur pipette through the air flow, activated by operating an external pedal linked to the stimulus air control. Each stimulus pipette was replaced after three consecutive recordings. 

### 2.4. Data Analysis

In the preliminary field assessment of DD food bait attractiveness, the mean weekly number and percentages of *D. suzukii* captures in each trap were obtained on the log/logit scale along with associated 95% confidence limits: these were back-transformed then divided by the number of days since trial set-up, to obtain the mean number of catches per trap per day. All analyses were carried out with Genstat [36]. 

The data obtained from the experimental work for the evaluation of the performance of *O. oeni* strains were analyzed by calculating the difference in the mean absorbance of bacterial growth. EAG recordings were analyzed by EAG software (EAG 2000 version 2.7, Syntech, Hilversum, Netherlands), and evaluated by measuring the maximum amplitude of negative deflection (-mVolt) obtained by stimulating the antenna. EAG amplitude values were compared across treatments by means of parametric one-way ANOVA, followed by Tukey’s test for the post hoc comparison of means. Homogeneity of variance had been previously determined with the Levene’s test. The statistical analyses were performed using Statistica, version 9 (Statsoft Inc., Tulsa, Oklahoma).

## 3. Results

### 3.1. Field Assessment of the Attractiveness of Droskidrink^®^ Food Baits Inoculated with Different Lactic Acid Bacteria

**Figure 1:**
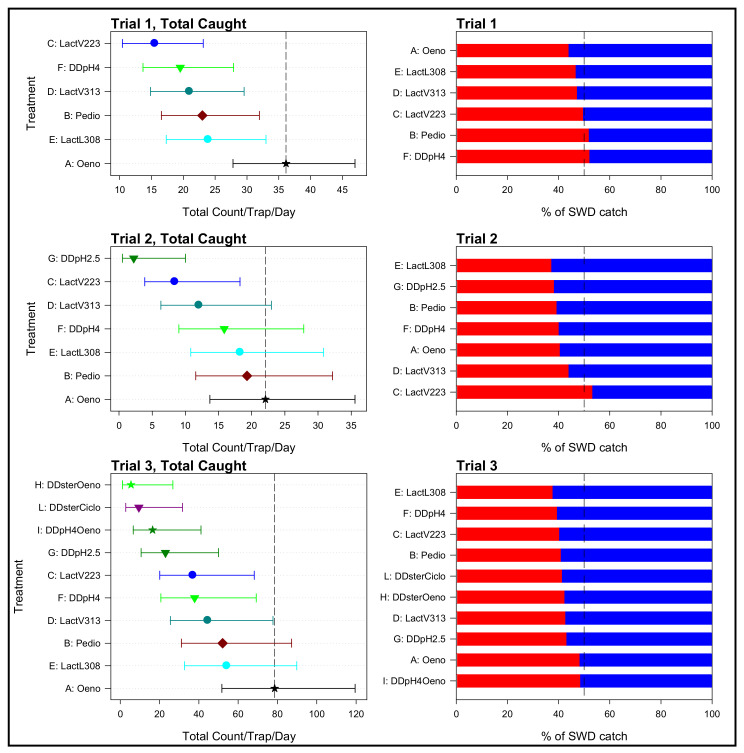
Left: Mean total SWD caught per trap per day for the three trials (including the final assessment, trial 3), with treatment in increasing order of the mean. Error bars are 95% confidence limits, and vertical dashed line is at the mean fortreatment A. Right: percentage of the total SWD that were female or male, for each of the three trials. Treatments are in increasing order of the % female. Dashed vertical line is at 50%. The red color indicates the percentage of caught females and the blue color indicates the percentage of caught males.

Figure 1 shows the total number of *D. suzukii* flies captured by different bait types during the preliminary field experiment. Generally, DD baits inoculated with the selected LAB species caught a significantly higher number of flies in comparison to the other DD baits (*p* < 0.001). Bait type A caught a significantly higher number of *D. suzukii* adults than baits inoculated with *Lactobacillus* spp. and *Pediococcus* spp. In three trials, there were significant differences in the numbers of males and total number of *D. suzukii* between the treatments (*p* = 0.027, 0.015, and 0.003 for males and *p* = 0.027, 0.017, and 0.001 for total *D. suzukii* for trials 1, 2, and 3, respectively) (Figure 1).

There was no significant difference in the numbers of females for trial 1 (*p* = 0.236), but there were significant differences in trials 2 and 3 (*p* = 0.023, 0.001, respectively). The percentage of female *D. suzukii* was similar for all treatments in trials 1 and 3 (*p* = 0.828, 0.061, respectively), but there was a significant difference between treatments in trial 2 (*p* = 0.039). Generally, the highest capture levels in all treatments were obtained in the fifth week of the experimental period and the capture peak corresponded to bait A.

The mean numbers of females were strongly correlated with the mean numbers of males (r = 0.95, 0.96, 0.95 for the three trials, respectively), reflecting that the patterns between the means were very similar for both sexes, and thus also similar to patterns in the total number of *D. suzukii* (Figure 1). For all three trials, the highest total number of *D. suzukii* was associated with bait A. Among the six baits tested first, bait type C had the lowest total number of *D. suzukii*, followed by bait type F. However, the total number of *D. suzukii* in bait type G was lower than in C, and for trial 3, the total number of *D. suzukii* in baits H, I, and L was lower than in bait G (Table S1). 

### 3.2. Assessment of the Performance of Oenococcus oeni Strains in Droskidrink^®^ Food Bait

All *O. oeni* strains, showed different growth rates in MRSm, with respect to the standard strains employed in the preliminary field experiment. The four considered variables (pH value 4.0, acetic acid concentration 45 g/L, ethanol content 4% (*v*/*v*), and incubation temperature 15 °C) allowed reasonable sorting of the *O. oeni* strains, according to the resistance to such limiting parameters.

**Figure 2:**
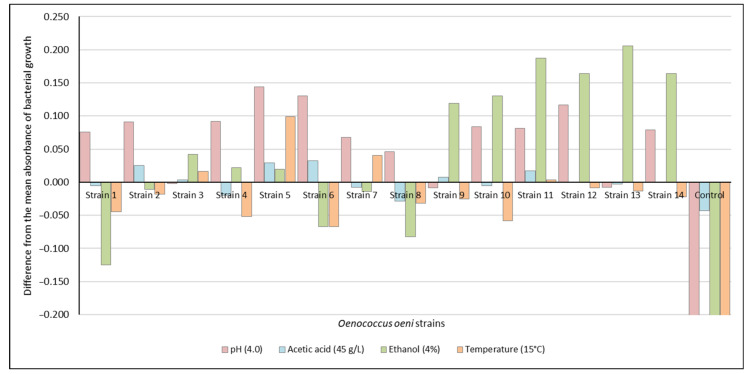
Differences in the mean absorbance of bacterial growth in synthetic media considering the four main DD limiting factors for *O. oeni* growth (pH 4.0), ethanol (4%), acetic acid (45 g/L), and temperature (15 °C).

Figure 2 shows the difference in the mean absorbance of *O. oeni* strains growth. Strain 3 showed the highest tolerance to low pH (4.0), and strain 14 had a significant tolerance to high acetic acid concentration (45 g/L). When compared with the control, strain 3 and strain 14 exhibited higher growth than the control at the tested pH and acetic acid concentration. Likewise, strain 2 showed the highest growth at high ethanol content (4%) and strain 12 showed the highest growth at low temperature (15 °C). In comparison with the control, growth of strain 2 and strain 12 was higher than the control at the tested ethanol content and temperature. Strain 2 and strain 12 showed high tolerance to high ethanol content (4%) and low temperature (15 °C), respectively. Overall, strain 2 and strain 12 showed a significant tolerance to the main limiting parameters for *O. oeni* growth, i.e., high ethanol content and low temperature. Strain 13 showed the highest tolerance to three limiting parameters, i.e., low pH, high acetic acid concentration, and low temperature (Table S2). Thus, the three strains selected for further experimentation for the evaluation of the behavioral responses of *D. suzukii* to volatiles emitted during fermentation process were strain 2, strain 12, and strain 13.

### 3.3. Electroantennography Responses of Drosophila suzukii Females to Volatile Collection of Oenococcus oeni Strains in Droskidrink^®^ Food Bait

**Figure 3:**
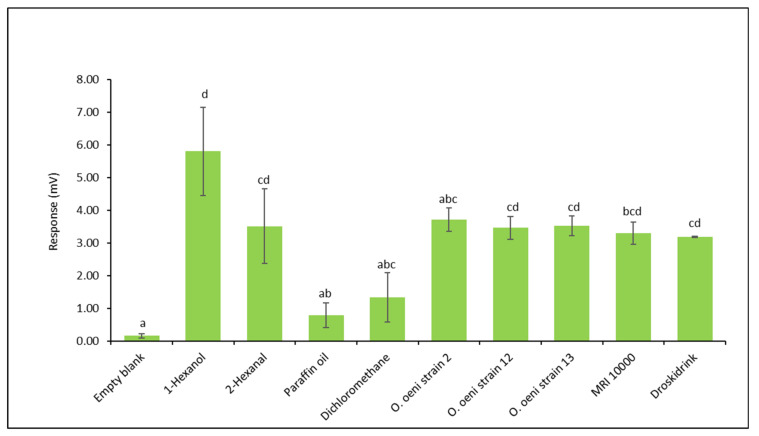
Mean electroantennography (EAG) responses (mV) of *D. suzukii* mated female antennae elicited by commercial DD and DD inoculated with different *O. oeni* strain 2, strain 12 and strain 13. Control stimuli: empty blank, paraffin oil, and dichloromethane solvent. Reference compounds: 1-hexanol, 2-hexanal. Baits: commercial DD, *O. oeni* reference strain (MRI 10000), strain 2, strain 12, and strain 13. The standard deviation of the means is reported. Bars represented by similar letters are significantly indifferent (*p* > 0.05).

The EAG responses of female *D. suzukii* to the headspace extracts obtained from DD inoculated with *O. oeni* strains are depicted in Figure 3. The reference compounds, 1-hexanol and 2-hexanal, elicited strong antennal responses (Figure S2). There were no statistical differences between the samples (DD, strain 2, 12 and 13) and the reference strain (MRI 10000), but all baits were statistically different from both the solvents and the blank control (ANOVA: d.f. = 47; F = 8.6; *p* < 0.001) (Table S3).

## 4. Discussion

The attractive effect of selected LAB species related to the biochemical changes of the bait such as the increase in the standard pH of DD (typically about 2.5) up to 4.0, since this value is recognized as ideal for the hetero-fermentative activity of wine LAB [37,38] was explored. Overall, the DD baits inoculated with the different LAB species attracted a considerably higher number of flies in comparison to other baits. The highest attractiveness was observed in the bait inoculated with *O. oeni*, which coincides with the outcomes of recent research comparing different lures for their attractiveness for *D. suzukii* in sweet cherry orchards [20]. On the contrary, pasteurized DD baits and DD bait mixed with tetracycline did not show any considerable attractiveness due to limited microbial activity in the liquid bait. 

Our findings imply that the utilization of the selected LAB species in DD bait was carried out successfully in the preliminary field trials. This is not surprising considering that they are an extremely important group of industrially related lactic bacteria, and considering their behavior and robustness under stressful conditions. LAB strains were used in our study to ferment the liquid bait and produce bioactive volatile compounds that are associated with *D. suzukii* attraction. Therefore, the type and concentration of volatiles present in the DD bait are greatly affected by the LAB strain used. Concerning the different species of LAB, it has been already mentioned that *O. oeni* have a remarkable hetero-fermentative activity and resistance in a low pH environment [37,38]. Therefore, their enhanced biological activity and effectiveness in trapping *D. suzukii* is expected. Recently, the addition of *O. oeni* to DD bait was evaluated in a comparative survey using Droso-Trap for monitoring fly populations in sweet cherry orchards. It was found that DD with *O. oeni* was effective in attracting *D. suzukii* during the blooming and until the beginning of fruit ripening [20]. These findings support our results, which show that *O. oeni* contributed to the highest attractiveness of the DD baits. Despite the differences in DD composition, field temperature, fruit phenological phases and pest population density between this comparative survey and our study, *O. oeni* in DD showed a similar trend in attracting flies, indicating a consistent fly response towards *O. oeni*. 

Furthermore, the obtained results show that the pH value in the liquid bait is a key point for the improvement of trap efficacy. In fact, the standard value of pH in DD, around 2.5, is unsuitable for the development of most LAB species, and thus the pH level has to be increased in order to provide them with an appropriate growing environment. On the other hand, too high pH values would promote a non-selective growth of contaminant microbes (yeasts, bacteria, or mold), causing a rapid depletion of the nutrients contained in the bait. 

Likewise, oenological trials showed that the pH value, adopted in the experiment, induces the shift of the metabolic activity of LAB from homo- to hetero-fermentation of sugars, increasing their energetic yield [37,38]. In our study, the selected LAB species were isolated from traditional Italian wine, indicating their ability as commercial starters to tolerate the stress conditions, i.e., acidic pH, encountered in the industrial winemaking process. However, several studies reported the ability of wine LAB to develop stress-induced responses through various mechanisms [39,40]. In earlier studies, *O. oeni* strains showed a high adaptive behavior to an acidic environment and a good malolactic performance at pH 3.5 and 3.0, respectively [31,40]. Similarly, *Lactobacillus* spp. and *Pediococcus* spp. exhibited acidophilic behavior at pH 3.0 and 3.5, respectively [41,42]. Under such stress conditions, the *Oenococcus, Lactobacillus,* and *Pediococcus* genera use the malolactic fermentation pathway to convert malic acid to lactic acid with the production of CO_2_. This pathway contributes to LAB survival enhancement and promotes growth [40]. In addition, it enhances their aroma profile, leading to a prevalence of the fruity aroma over the vegetative ones [43]. Based on the previous findings, it is obvious that the LAB tested in our study were able to tolerate low pH and to produce fermentative volatiles that mediate fly attraction and significantly contribute to improving the attractiveness of the DD bait. Among all the tested LAB, *O. oeni* with DD was the most attractive bait. Overall variations in capturing levels between the tested LAB could be related to environmental conditions that may affect the fermentation process and, consequently, the volatile profiles qualitatively and quantitatively. The non-linear increase in trap catches over the time of field exposure could be explained by the fact that the traps were only refilled every week to compensate the bait evaporation and hence the liquid bait was not completely replaced during the entire duration of the field test in order to maintain the original population of bacteria inside it. 

In our field trials, it was observed that DD baits with LAB attracted *D. suzukii* flies in a species- and strain-dependent manner in the first week. The following three weeks showed a fluctuation and a decrease in captured flies. Thereafter, capture peaks were found in all DD baits with LAB in the fifth week. These sharp variations in the weekly captures can be related to several factors affecting the LAB metabolic activities. The high concentration of LAB (10^6^ CFU/mL) in the first week may help to maintain their metabolic activities and production of volatiles that mediate fly’s attraction [44]. In the subsequent weeks, it is anticipated that stress conditions in DD bait have a negative impact on LAB growth and metabolism, resulting in low attraction levels. As part of the stress response, LAB develop an adaptive response which enables them to survive and maintain their metabolic activities. During the fermentation process, the LAB can produce a series of biologically active compounds that affect fruit flies [45]. Consequently, volatile compounds that are most prevalent in the main components of DD (wine and vinegar), i.e., methanol, ethanol, acetic acid, and ethyl acetate, will be enriched by LAB fermentation. The results and observations reported in our study clearly suggest that the tested LAB strains contribute to improving the attractiveness of a commercial lure (DD) and recognize them as suitable agents for a more effective lure. *O. oeni*, the best-adapted species to the stress conditions [40], can be exploited as pest control agent in eco-friendly and sustainable management strategies.

In the laboratory evaluation of *O. oeni* performance in DD conditions, the addition of bacteria to DD as bio-catalyzers for the production of bioactive volatile compounds is completely new. Therefore, it is necessary to evaluate the performance of *O. oeni* strains under stress conditions in DD bait. The four main DD characteristics (low pH, high ethanol content, high acetic acid concentration, and low temperatures during field application) that can limit the growth of *O. oeni* strains were tested. The outcomes are particularly interesting in view of the double effect of acetic acid on experimental conditions. In addition, *O. oeni* was able to lower the pH level (2.5), as an end-product of many of its metabolic activities [37], and it can inhibit bacterial activity, affecting the ratio between substrates consumption and metabolites production. 

The laboratory tests made it possible to identify three *O. oeni* strains that exhibited different adaptation to the peculiar DD composition. These strains were tested for their ability to produce volatile compounds, by the fermentative metabolism in DD, with a biological activity on *D. suzukii*. However, the relevant difference among the three *O. oeni* strains required further field tests in order to select the most attractive bait for *D. suzukii* monitoring.

In EAG analysis, 1-hexanol was already detected among the volatile bouquet of fresh mature *D. suzukii* host fruits and elicited fly’s antennal responses in gas chromatographic analysis coupled with electroantennographic detection experiments (GC-EAD) [35,46]. Moreover, EAG analysis confirmed remarkable insect sensitivity to both DD and DD inoculated with *O. oeni*. Additional GC-EAD experiments were performed [47] to understand which single compounds can be perceived by the olfactory system of *D. suzukii*. The selection of single biologically active compounds may pave the way to the development of synthetic attractants.

To better explain *D. suzukii* preference for DD baits inoculated with *O. oeni* strains, a recent work identified the volatiles profile of the various strains and determines their essential role in fly’s preferences [47]. Although it was not possible to identify an overall attraction pattern for *O. oeni* strains, the study showed that three *O. oeni* strains produced the most attractive volatiles. A combination of the most effective strains was used to optimize trap efficiency for pest management. To get a deeper insight into their nature, fly responses to the new DD baits using different trap designs were determined in open field conditions [47]. Such field trials will be exploited to develop a new, efficient trapping system for *D. suzukii* management.

## 5. Conclusions

The study demonstrates that different strains of the *O. oeni* species can improve the attractiveness of the commercial food bait DD, making it a powerful tool for pest control and monitoring. The outcomes of a series of preliminary field trials and laboratory tests highlight the importance of further investigations of the chemical and microbiological properties of DD with regard to the mechanisms of fly’s attraction, in order to produce a highly attractive bait for pest management. 

Additionally, the findings of this study pave the way for the development of a new trapping concept, in which the attractiveness of DD would be improved by the use of LAB volatiles in a strain-dependent manner. Thus, further laboratory and open field trials on the chemical characterization of volatiles produced in baits inoculated with *O. oeni* strains, providing the optimal bacterial growth in open field conditions, are useful for the successful setup of new efficient traps.

## Figures and Tables

**Table 1:** Composition of Droskidrink^®^ food baits tested in a preliminary field experiment in a vineyard var. Teroldego in San Michele all’Adige, Trento, Italy.

Type	Composition
**Bait A**	DD (pH 4.0) inoculated with *Oenococcus oeni* ATCCBAA-331
**Bait B**	DD (pH 4.0) inoculated with *Pediococcus* spp.
**Bait C**	DD (pH 4.0) inoculated with *Lactobacillus* spp. V223
**Bait D**	DD (pH 4.0) inoculated with *Lactobacillus* spp. V313
**Bait E**	DD (pH 4.0) inoculated with *Lactobacillus* spp. L308
**Bait F**	DD at pH 4.0
**Bait G**	DD at pH 2.5 (commercial version)
**Bait H**	DD at pH 2.5 pasteurized at 70 °C for 30 min and inoculated with *Oenococcus oeni*
**Bait I**	DD at pH 4.0 pasteurized at 70 °C for 30 min, mixed with 10 mL/L of cycloheximide aqueous solution (0.01%), and inoculated with *Oenococcus oeni*
**Bait L**	DD at pH 4.0 mixed with 1 g of tetracycline and inoculated with *Oenococcus oeni*

## Data Availability

The data presented in this study are available on request from the corresponding author.

## Supplementary Materials

The following are available online at https://www.mdpi.com/2075-4450/12/2/153/s1, Figure S1: Layout and duration of field trials, Figure S2: Electroantennogram plots recorded from SWD antenna, in response to volatile collection of *O. oeni* strains in Droskidrink^®^ Bait., Table S1: Field assessment of attractiveness of Droskidrink^®^ baits inoculated with lactic acid bacteria, Table S2: Assessment of *O. oeni* strains’ growth in Droskidrink^®^ bait, Table S3: Electroantennography responses of SWD females to volatile collection of *O. oeni* strains in Droskidrink^®^ Bait.
