# Supplementary material for: Selection of Lactic Acid Bacteria Species and Strains for Efficient Trapping of Drosophila suzukii
Source: Insects. 2021 Feb 11;12(2):153. doi: 10.3390/insects12020153 (PMC7918454; doi:10.3390/insects12020153)
Supplement: Supplementary file 1 [file insects-12-00153-s001.pdf]

**Trial 1** (16 September –9 October 2013)									
A1	B1	C1	D1	E1	F1				
E2	C2	D2	F2	B2	A2				
C3	F3	B3	A3	E3	D3				
**Trial 2** (9 October –23 October 2013)									
A1	B1	C1	D1	E1	F1	G1			
G2	E2	C2	D2	F2	B2	A2			
C3	F3	B3	A3	E3	D3	G3			
**Trial 3** (23 October –30 October 2013)									
A1	B1	C1	D1	E1	F1	G1	H1	I1	L1
L2	I2	H2	G2	E2	C2	D2	F2	B2	A2
H3	C3	L3	F3	B3	A3	E3	D3	I3	G3

Figure S1: Layout and duration of field trials comparing attractiveness of Droskidrink food baits in a commercial vineyard.

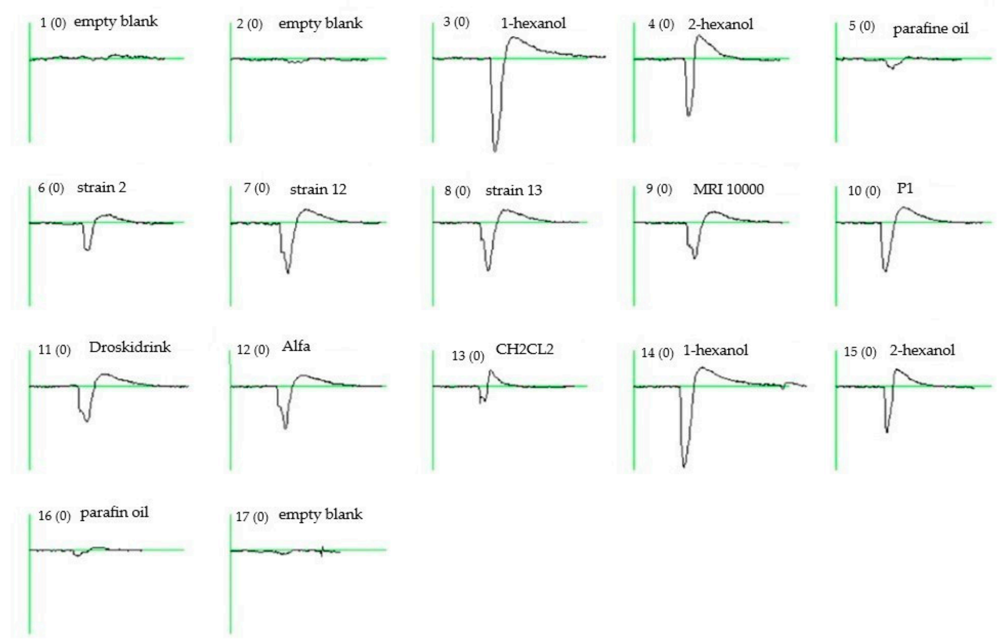

Figure S2: Electroantennogram plots recorded from SWD antenna, in response to volatile collection of *O. oeni* strains in Droskidrink® Bait. Control stimuli: empty blank, paraffin oil, and dichloromethane solvent. Reference compounds: 1-hexanol, 2-hexanal. Baits: commercial DD, *O. oeni* reference strain (MRI 10000), strain 2, strain 12, and strain 13.

Trial	Treat	Male	Female	Total	%female
1	A	20.2 (15.0,27.2)	15.9 (11.2,22.6)	36.1 (27.8,47.0)	44.0 (35.1,53.3)
	B	11.0 (7.4,16.5)	12.0 (8.0,17.9)	23.0 (16.6,31.9)	52.0 (40.4,63.3)
	C	7.8 (4.8,12.6)	7.7 (4.7,12.7)	15.5 (10.4,23.1)	49.7 (35.9,63.5)
	D	11.0 (7.4,16.5)	9.9 (6.4,15.4)	21.0 (14.9,29.6)	47.3 (35.5,59.4)
	E	12.7 (8.7,18.5)	11.2 (7.4,17.0)	23.9 (17.3,33.0)	46.8 (35.8,58.2)
	F	9.3 (6.0,14.5)	10.2 (6.6,15.8)	19.5 (13.7,27.9)	52.2 (39.7,64.4)
2	A	13.1 (8.0,21.6)	9.0 (5.6,14.3)	22.1 (13.7,35.6)	40.6 (36.3,45.0)
	B	11.7 (6.9,19.8)	7.6 (4.6,12.6)	19.3 (11.6,32.2)	39.3 (34.8,44.1)
	C	3.9 (1.6,9.8)	4.5 (2.3,8.7)	8.4 (3.9,18.2)	53.3 (46.1,60.4)
	D	6.7 (3.4,13.5)	5.3 (2.9,9.7)	12.0 (6.3,23.0)	44.0 (38.2,50.0)
	E	11.4 (6.7,19.5)	6.8 (4.0,11.7)	18.3 (10.8,30.9)	37.3 (32.7,42.1)
	F	9.5 (5.3,17.0)	6.4 (3.7,11.1)	15.9 (9.0,27.8)	40.2 (35.2,45.4)
	G	1.4 (0.3,6.4)	0.9 (0.2,3.9)	2.2 (0.5,10.0)	38.3 (25.9,52.5)
3	A	40.6 (25.4,64.8)	38.0 (26.1,55.3)	78.6 (51.7,119.5)	48.3 (44.6,52.1)
	B	30.7 (17.9,52.6)	21.4 (13.0,35.3)	52.1 (31.1,87.2)	41.0 (36.6,45.6)
	C	22.0 (11.7,41.6)	15.0 (8.2,27.2)	37.0 (20.1,68.2)	40.4 (35.2,45.9)
	D	25.5 (14.1,46.0)	19.0 (11.2,32.4)	44.5 (25.5,77.7)	42.8 (37.9,47.8)
	E	33.8 (20.2,56.4)	20.5 (12.3,34.2)	54.2 (32.7,89.8)	37.8 (33.5,42.2)
	F	22.9 (12.3,42.7)	15.0 (8.2,27.2)	37.8 (20.7,69.2)	39.5 (34.4,44.9)
	G	13.1 (5.7,29.8)	10.0 (4.8,20.8)	23.0 (10.6,49.9)	43.2 (36.5,50.2)
	H	3.2 (0.6,16.9)	2.3 (0.5,10.6)	5.5 (1.1,26.8)	42.4 (29.3,56.7)
	I	8.5 (3.1,23.5)	8.0 (3.5,18.1)	16.5 (6.6,41.1)	48.6 (40.5,56.7)
	L	5.5 (1.6,19.7)	3.9 (1.2,12.7)	9.5 (2.8,31.7)	41.5 (31.3,52.4)

Table S1: Field assessment of attractiveness of Droskidrink® baits inoculated with lactic acid bacteria. Number of SWD caught per trap per day, for each of the treatments for three successive trials (95% confidence limits).

	pH (4.0)	Acetic acid (45 g/l)	Ethanol (4%)	Temperature (15°C)
Strain 1	0.075	-0.005	-0.125	-0.044
Strain 2	0.091	0.025	-0.011	-0.018
Strain 3	-0.002	0.003	0.042	0.017
Strain 4	0.092	-0.020	0.022	-0.052
Strain 5	0.144	0.029	0.019	0.099
Strain 6	0.130	0.032	-0.067	-0.067
Strain 7	0.068	-0.008	-0.014	0.040
Strain 8	0.046	-0.029	-0.083	-0.032
Strain 9	-0.008	0.007	0.119	-0.026
Strain 10	0.083	-0.005	0.130	-0.058
Strain 11	0.081	0.017	0.187	0.003
Strain 12	0.117	-0.001	0.164	-0.009
Strain 13	-0.008	-0.003	0.205	-0.014
Strain 14	0.079	0.000	0.164	-0.022
Control	-0.990	-0.043	-0.755	-1.403

Table S2: Assessment of *O. oeni* strains' growth in Droskidrink® bait. Differences in the mean absorbance of bacterial growth in MRSm medium considering the main DD limiting parameters for *O. oeni* growth (pH 4.0), and ethanol (4%), acetic acid (45 g/L) at temperature (15 °C).

Samples	Mean	SDV
Empty blank	0.15	0.06
1-Hexanol	5.80	1.35
2-Hexanal	3.51	1.14
Paraffin oil	0.79	0.37
Dichloromethane	1.33	0.76
O. oeni strain 2	3.71	0.36
O. oeni strain 12	3.46	0.35
O. oeni strain 13	3.52	0.30
MRI 10000	3.30	0.34
Droskidrink	3.19	0.02

Table S3: Electroantennography responses of SWD females to volatile collection of *O. oeni* strains in Droskidrink® Bait. Mean responses (mV) of mated female antennae elicited by commercial DD and DD inoculated with different *O. oeni* strain 2, strain 12 and strain 13. Control stimuli: empty blank, paraffin oil, and dichloromethane solvent. Reference compounds: 1-hexanol, 2-hexanal. Baits: commercial DD, *O. oeni* reference strain (MRI 10000), strain 2, strain 12, and strain 13. The standard deviation of the means is reported.
